# Transcriptome Profiling of Acquired Gefitinib Resistant Lung Cancer Cells Reveals Dramatically Changed Transcription Programs and New Treatment Targets

**DOI:** 10.3389/fonc.2020.01424

**Published:** 2020-08-14

**Authors:** Nan Wei, Yong'an Song, Fan Zhang, Zhifu Sun, Xiaoju Zhang

**Affiliations:** ^1^Department of Respiratory and Critical Care Medicine, Henan Provincial People's Hospital, Zhengzhou University People's Hospital, Zhengzhou, China; ^2^Academy of Medical Science, Zhengzhou University, Zhengzhou, China; ^3^Department of Health Sciences Research, Mayo Clinic, Rochester, MN, United States

**Keywords:** lung adenocarcinoma, EGFR mutation, gefitinib, drug resistance, RNA sequencing, KPT-185, dasatinib

## Abstract

**Background:** Targeted therapy for lung cancer with epidermal growth factor receptor (*EGFR*) mutations with tyrosine kinase inhibitors (TKIs) represents one of the major breakthroughs in lung cancer management. However, gradually developed resistance to these drugs prevents sustained clinical benefits and calls for resistant mechanism research and identification of new therapeutic targets. Acquired T790M mutation accounts for the majority of resistance cases, yet transcriptome changes in these cells are less characterized, and it is not known if new treatment targets exist by available drugs.

**Methods:** Transcriptome profiling was performed for lung cancer cell line PC9 and its resistant line PC9GR after long-term exposure to gefitinib through RNA sequencing. Differentially expressed genes and changed pathways were identified along with existing drugs targeting these upregulated genes. Using 144 lung cancer cell lines with both gene expression and drug response data from the cancer cell line encyclopedia (CCLE) and Cancer Therapeutics Response Portal (CTRP), we screened 549 drugs whose response was correlated with these upregulated genes in PC9GR cells, and top drugs were evaluated for their response in both PC9 and PC9GR cells.

**Results:** In addition to the acquired T790M mutation, the resistant PC9GR cells had very different transcription programs from the sensitive PC9 cells. Multiple pathways were changed with the top ones including TNFA signaling, androgen/estrogen response, P53 pathway, MTORC1 signaling, hypoxia, and epithelial mesenchymal transition. Thirty-two upregulated genes had available drugs that can potentially be effective in treating the resistant cells. From the response profiles of CCLE, we found 17 drugs whose responses were associated with at least four of these upregulated genes. Among the four drugs evaluated (dasatinib, KPT-185, trametinib, and pluripotin), all except trametinib demonstrated strong inhibitory effects on the resistant PC9GR cells, among which KPT185 was the most potent. KPT-185 suppressed growth, caused apoptosis, and inhibited migration of the PC9GR cells at similar (or better) rates as the sensitive PC9 cells in a dose-dependent manner.

**Conclusions:** Acquired TKI-resistant lung cancer cells (PC9GR) have dramatically changed transcription and pathway regulation, which expose new treatment targets. Existing drugs may be repurposed to treat those patients with developed resistance to TKIs.

## Introduction

Lung cancer is the leading cause of cancer deaths around the world with staggering 15% of five-year survival rates. Non-small cell lung cancer (NSCLC), consisting mainly of lung adenocarcinoma and squamous cell carcinoma, represents about 85% of all lung cancer cases (1). The most effective treatment is surgical removal for early stage NSCLC; however, most patients are diagnosed at late stages when the mainstays of treatments are chemotherapy and radiotherapy. Due to the side effects and low efficacy of these treatments, targeted therapies for patients with certain gene mutations have become an appealing alternative (2), among which mutations in *EGFR* and *EML4-ALK* translocation are two typical examples and routinely tested to guide clinical treatment selections. Both *EGFR* and *EML4-ALK* mutations are mainly seen in lung adenocarcinoma, and these activated oncogenic mutations promote cell survival, proliferation, invasion, and metastasis (3, 4). Abolishment of their activities through TKIs may inhibit tumor growth and lead to tumor shrinkage and complete response in some of the patients with EGFR mutations (5–8). The major challenge in the clinic, however, is gradually developed resistance to these TKIs (9, 10), and an alternative drug targeting new mutations or a next-generation TKI is generally needed to maintain treatment effectiveness. Understanding the mechanism of acquired resistance is critical to identify new targets and develop new treatment strategies.

Several TKI-resistant mechanisms have been proposed. It has been observed that 50–60% of those with subsequent TKI resistance develop a secondary mutation T790M (10–13). Other acquired single nucleotide mutations include D761Y, T854A, and L747S in *EGFR* (14, 15). Gene amplification is also reported for *MET* (16, 17), *HER2* (18), and *MAPK* (19). For tumors without acquired or primary resistant mutations, abnormal epigenetic regulation may be in play (20, 21). Resistant tumors may have an epithelial-to-mesenchymal transition (EMT) phenotype with accompanying high expression of vimentin or fibronectin (22–24) or N-cadherin (25); *AXL* or *GAS6* activation promotes cell proliferation, migration, and invasion in cancer (26, 27); and activated NF-κB pathway (28) and IGF1-R pathway are also reported with TKI resistance (29).

Although T790M mutation is the major resistant mechanism, transcriptome changes in these cells are not well-characterized. We hypothesized that the resistant cells had very different transcription programs and may expose new treatment targets with existing drugs to overcome the resistance. To test the hypothesis, we used an *EGFR*-mutant PC9 lung cancer cell line that is highly sensitive to TKIs initially and developed a stable drug-resistant cell line PC9GR after long-term exposure to gefitinib. Through an RNA sequencing (RNA-seq) experiment, we profiled the genetic and transcriptome differences between the resistant and sensitive cell lines to getitinib. The acquired resistant cell line PC9GR obtained the well-known T790M mutation, but more noticeably, it had dramatically changed transcriptome programs and regulation pathways. Combined with CCLE gene expression and drug response data, we have identified several available drugs that can be potentially used to treat the resistant cells or tumors.

## Materials and Methods

### Cell Lines

Human lung cancer PC9 and PC9GR cell line generation were described in our previous work (30). The PC9 was derived from lung adenocarcinoma and is inherently sensitive to TKIs, such as gefitinib and erlotinib. Genetically, it harbors a canonical 15 base deletion at exon19 of EGFR. The established resistant PC9GR was obtained after long-term exposure to gefitinib with over 260-fold higher resistance than its parental PC9 cells.

Both PC9 and PC9GR were verified for their authentication with ATCC PC9 profile through STR test using nine markers (Shanghai Biowing Biotechnology, Shanghai, China).

### RNA Sequencing and Data Analysis

The library preparation, sequencing, and initial data preprocessing were described previously (30). Quantified raw gene count was normalized through fragments per kilobase million (FPKM) and then log2 transformed for further analysis. Differential expression between PC9GR and its parenting PC9 cells was conducted by R package limma (31). Genes that have a false discovery rate (FDR) <0.05, log2 fold change >1 (2-fold), and minimum mean expression across all samples (in FPKM) >1 were considered as differentially expressed genes (DEGs). Pathway or gene set enrichment analysis was performed for these DEGs by rapid integration of term annotation (RITAN) (32), and those with FDR >0.05 were considered as significantly enriched. As HiSAT2 does not map sequence reads with short insertion/deletion (indels) well (33), we further performed two-step alignment by spliced transcript alignment to a reference (STAR) (34), and the alignment from this was used for indel detection.

### Identification of Drugs That Target Upregulated Key Genes in PC9GR-Resistant Cells

To identify existing drugs that can potentially be used to treat gefitinib-resistant lung cancer cells, we used ingenuity pathway analysis (IPA) gene annotations for which an available drug was provided to select the DEGs that were unregulated in PC9GR cells. To get more evidence of an available drug that may indeed be effective and act on the intended target gene, we further analyzed the gene expression and drug response data from CCLE (https://portals.broadinstitute.org/ccle/about), and CTRP (https://portals.broadinstitute.org/ctrp). Processed and normalized RNA sequencing data for 1,076 cell lines in RPKM were downloaded, and then data for lung cancer cell lines (*n* = 189) were extracted. Drug response data for 545 drugs and 886 cell lines were downloaded, and lung cancer cell lines with both RNA-seq and drug response data (*n* = 144) were used for correlation analysis between expression of DEGs that were upregulated and with drugs whose response data were tested in CTRP. The drug and gene pairs with correlation coefficient <-0.3 and significant *p* < 0.001 were kept for further investigation.

### Evaluation of Treatment Response to Selected Drugs

#### Cell Viability Assay

PC9 and PC9GR cells in logarithmic growth stage were seeded in 96-well plates at a density of 3,000 cells per well and grown overnight. The next day, the growth medium was replaced with fresh media with dasatinib (MedChemExpress, Monmouth Junction, NJ, USA), pluripotin (MedChemExpress, Monmouth Junction, NJ, USA), trametinib (MedChemExpress, Monmouth Junction, NJ, USA), and KPT-185 (MedChemExpress, Monmouth Junction, NJ, USA), respectively, by the gradient dilution method. After being incubated for 72 h, Cell Counting Kit 8 (APExBIO, Houston, Texas, USA) was added for an additional 2 h of incubation at 37°C. Cell viability was determined by measuring the absorbance at 450 nm in a microplate reader (Thermo, Waltham, MA, USA).

### Colony Formation Assay

PC9 and PC9GR cells in logarithmic growth stage were seeded in six-well plates at a density of 3,000 cells per well and grown overnight. The next day, the growth medium was replaced with fresh media with multiple dilution concentrations of KPT-185 at 37°C for 9 days. The medium was then discarded, washed with PBS three times, and fixed with 4% paraformaldehyde for 2 h. After staining with 0.1% crystal violet for 30 min, the colonies were visualized and photographed.

### Flow Cytometric Apoptosis Assay

PC9 and PC9GR cells in logarithmic growth stage were seeded in six-well plates at a density of 2 × 10^5^ cells per well and grown overnight. The next day, the growth medium was replaced with fresh media with multiple dilution concentrations of KPT-185 at 37°C for 48 h. The cells from both suspension and adherence were collected and resuspended in binding buffer containing Annexin V-fluorescein isothiocynate (FITC). Staining solution with propidium iodide (PI) was then added following the kit instructions, and localization of Annexin V and PI for apoptotic cells was performed by FACS cytometry (BD Biosciences, Franklin Lakes, NJ, USA) and percentage of apoptotic cells were obtained.

### Wound Healing Assay

PC9 and PC9GR cells in logarithmic growth stage were seeded in six-well plates at a density of 4 × 10^5^ cells per well and grown overnight. The next day, the cell layer was wounded with a yellow pipette tip, and floating cells were washed with PBS. Then, 2% FBS medium containing KPT-185 (0.25, 0.5, 1, and 2 μM) was added into each well. After incubating for 0 and 48 h, three randomly chosen fields were analyzed for each well, and cell migration rate was calculated relative to control well-without KPT-185.

All data analyses were conducted in R 3.5.2 (https://www.r-project.org/) or otherwise stated. The raw and processed RPKM data was deposited into GEO with accession number GSE129221 (https://www.ncbi.nlm.nih.gov/geo/query/acc.cgi?acc=GSE129221).

## Results

### Phenotypic Characteristics of PC9 Gefitinib-Resistant Cells

**Figure 1:**
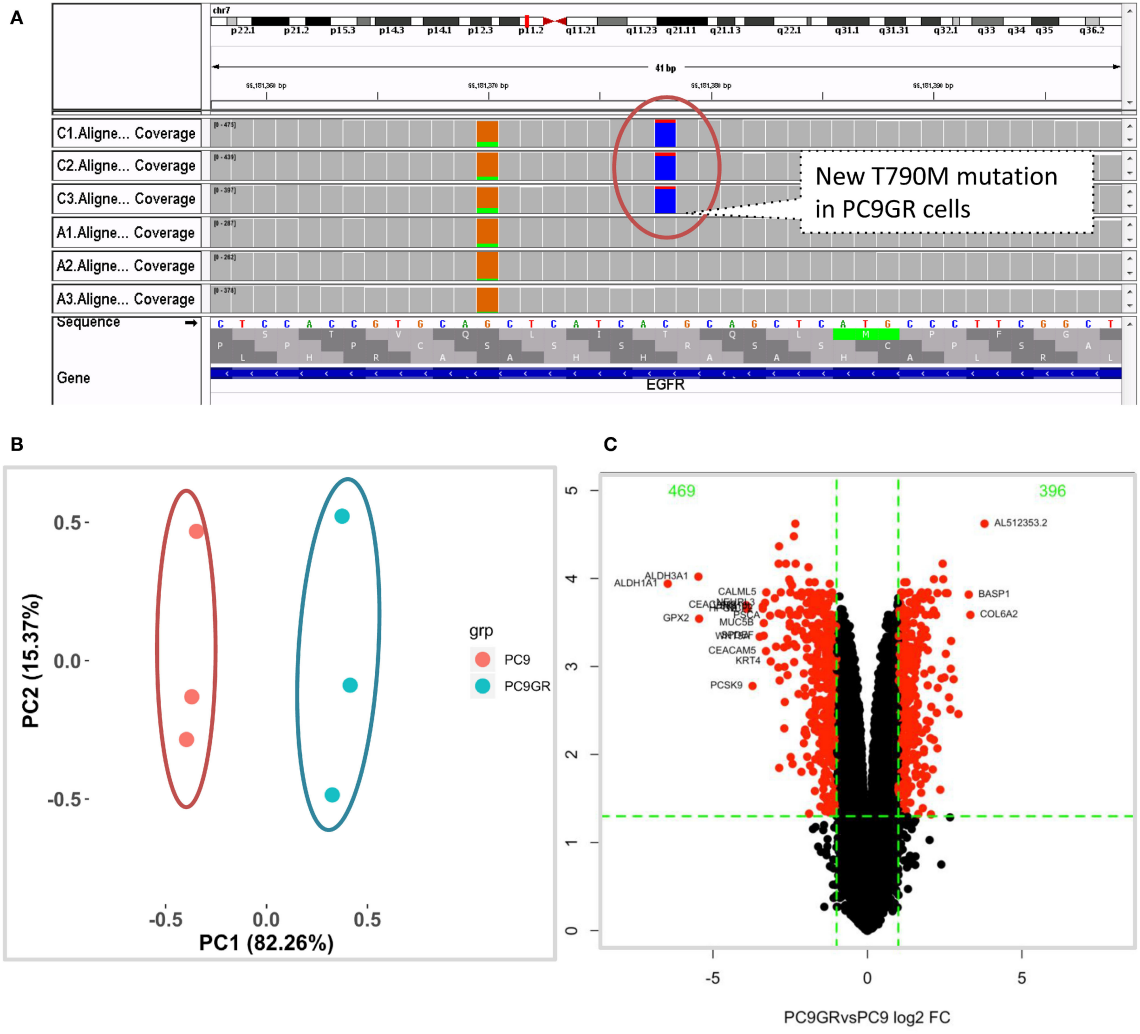
Mutation and gene expression profiles of PC9 and resistant PC9GR cells. **(A)** IGV view of EGFR gene from RNA-seq sequence reads showing acquired T790M mutation in the PC9GR cells (marked as C1, C2, C3 in the IGV tracks), which is absent in parental PC9 cells (marked as A1, A2, A3). **(B)** Principal component analysis for PC9 and PC9GR cells. Component 1 accounts for 82% of variance, and resistant cells have quite different expression profiles from PC9 cells. **(C)** Volcano plot of differential expression analysis between PC9GR and PC9 cells. Red highlights are genes with false discovery rate <0.05 and fold change >2 (either higher or lower in PC9GR cells). The green numbers on the top are the number of down (469) and up (396) regulated genes in PC9GR cells.

After 1 year of gefitinib treatment, we successfully established a gefitinib-resistant NSCLC cell line PC9GR from the gefitinib-sensitive PC9 cell line. The IC50 value for gefitinib in PC9GR cells was 5.311 ± 0.455 μM, which is 265-fold higher than that in PC9 cells (0.020 ± 0.003 μM) as we previously reported (30). From RNA-seq of these cell lines, we confirmed that both PC9 and PC9GR cells had 15-base deletion on exon 19 of EGFR. However, the PC9GR cells obtained a newly developed T790M mutation (Figure 1A), mimic to the clinical observation that an initial sensitive tumor develops resistance to gefitinib. This mutation is known to be the most common reason in the clinic for patients who develop TKI resistance after treatment (10, 35, 36). No other reported mutations reported previously (D761Y, T854A, and L747S) (14, 15) were found in the PC9GR cells.

### Distinct RNA Expression Profiles Between PC9GR and PC9 Cells

**Figure 2:**
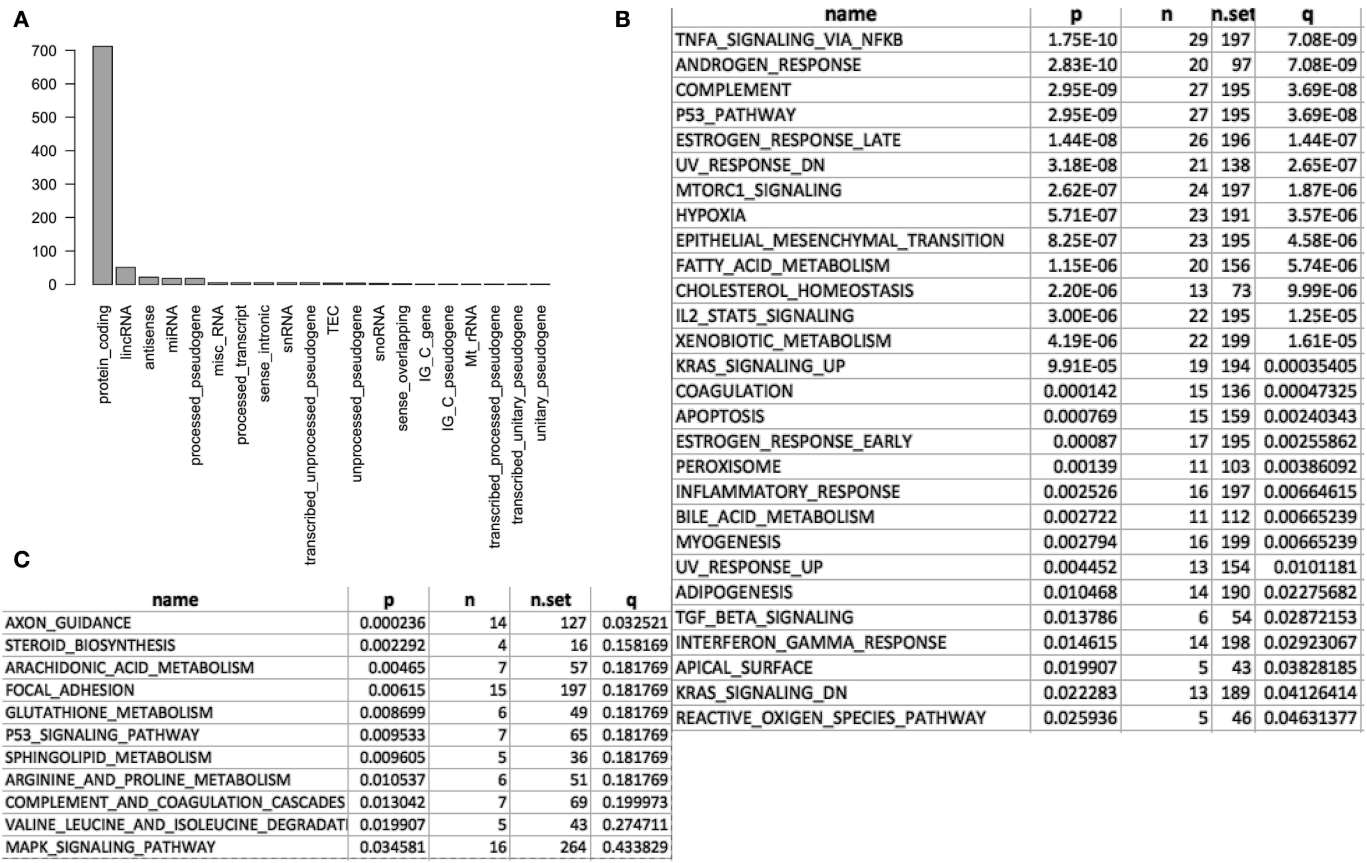
Differentially expressed genes (DEG) and enriched gene sets and pathways. **(A)** DEG distribution by gene class. Protein coding is the most and lincRNA is the second. **(B)** Significantly enriched gene sets from MSigDB. **(C)** Significantly enriched KEGG pathways.

**Figure 3:**
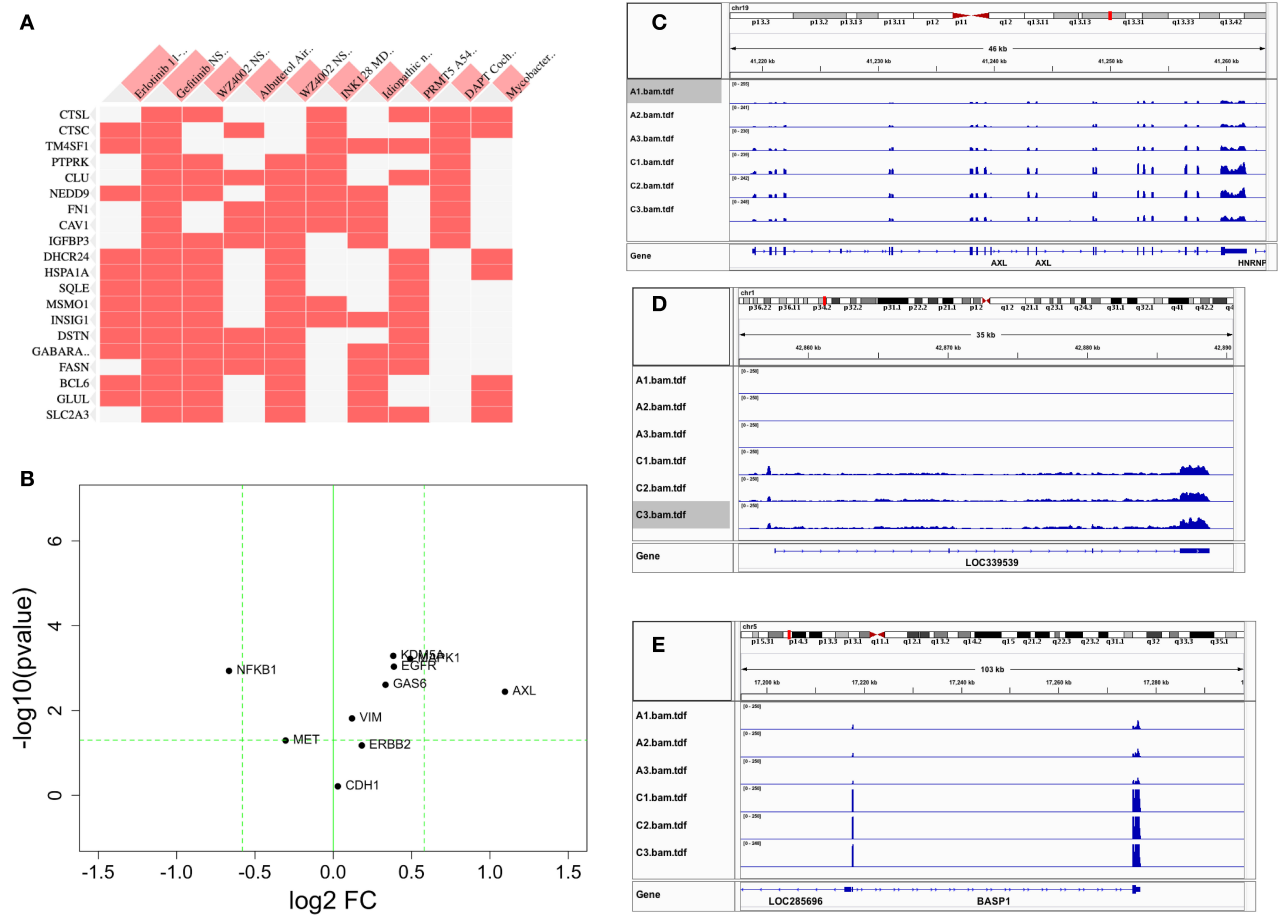
Comparison of differentially expressed genes with those reported in the literature. **(A)** Heat map of DEGs vs. other reports (38) or GEO databases (GSE75602, GSE65420, GSE73189). Genes on the row are DEGs from this study, and columns are gene sets reported from other studies. The first two columns are the most relevant, the genes upregulated after drug resistance to Erlotinib and Gefitinib. **(B)** The change of reported genes from the literature [*AXL/GAS6* (26, 27)*, CDH1* (39)*, ERBB2* (18)*, MAPK1* (19), *MET* (16)*, VIM* (22–24)*, NFKB1* (28), and *KDM5A* (29)] in our study. X-axis is log2 fold change between PC9GR vs. PC9. Y-axis is negative log10 *p*-value. Horizontal dashed line is *p* value 0.05, and two vertical dashed lines are for fold change less and larger than 1.5-fold, respectively. Only AXL is dramatically up-expressed. **(C–E)** are IGV view of gene expression for *AXL, LOC339539*, and *BASP1*. Sample names A1-A3 in the IGV tracks are parental PC9 cells and C1-C3 are for PC9GR cells.

We first conducted principal component analysis (PCA) for the RNA-seq samples from PC9 and PC9GR cells and found that they formed very distinct clusters, and the first principal component explained over 82% of variance, a clear indication that they had very different expression profiles in addition to the T790M mutation (Figure 1B). Differential expression analysis identified 865 DEGs (396 up- and 469 down-expressed, Figure 1C and Table S1) at FDR < 0.05, log2 fold change >1, and minimum mean expression across samples in FPKM > 1. The vast majority of these DEGs were protein-coding genes (712 out of 865, 82.3%); however, there were many other differentially expressed RNAs, particularly lncRNAs and pseudogenes (Figure 2A). Twenty-eight MSigDB pathways were significantly enriched at FDR < 0.05 although only one was in the Kyoto Encyclopedia of Genes and Genomes (KEGG) pathways at this stringent cutoff (Figures 2B,C). The most notable ones were TNFA_signaling_via_NFKB, androgen/estrogen response, P53_pathway, MTORC1_signaling, hypoxia and epithelial_mesenchymal_transition. NF-κB is reported to be activated early in response to *EGFR* targeted therapy by TKIs (37). Epithelial-to-mesenchymal transition is known to be involved in late obtained T790M-associated drug-resistant PC9 cells (PC9GR3) (38). Many of these DEGs are among the upregulated genes previously reported after resistance development to erlotinib (37) or gefitinib (38) (Figure 3A).

Previous studies reported that *AXL/GAS6* (26, 27)*, CDH1* (39)*, ERBB2* (18)*, MAPK1* (19), *MET* (16)*, VIM* (22–24)*, NFKB1* (28), and *KDM5A* (29) were increased in TKI-resistant cells. In these data, we only found *AXL* was increased dramatically with 2.14-fold change and FDR < 0.05. *MAPK1, VIM, GAS6, NFKB1, and KDM5A* were all slightly increased, and *CDH1, ERBB2*, and *MET* had not much change (Figure 3B). As a contrast to *AXL* (Figure 3C), the top two upregulated genes *AL512353.2* (also named LOC339539, Figure 3D) and *BASP1* (Figure 3E) had over 8-fold increase.

### Candidate Drugs That Can Potentially Be Effective in Treating Resistant PC9GR Cells

**Figure 4:**
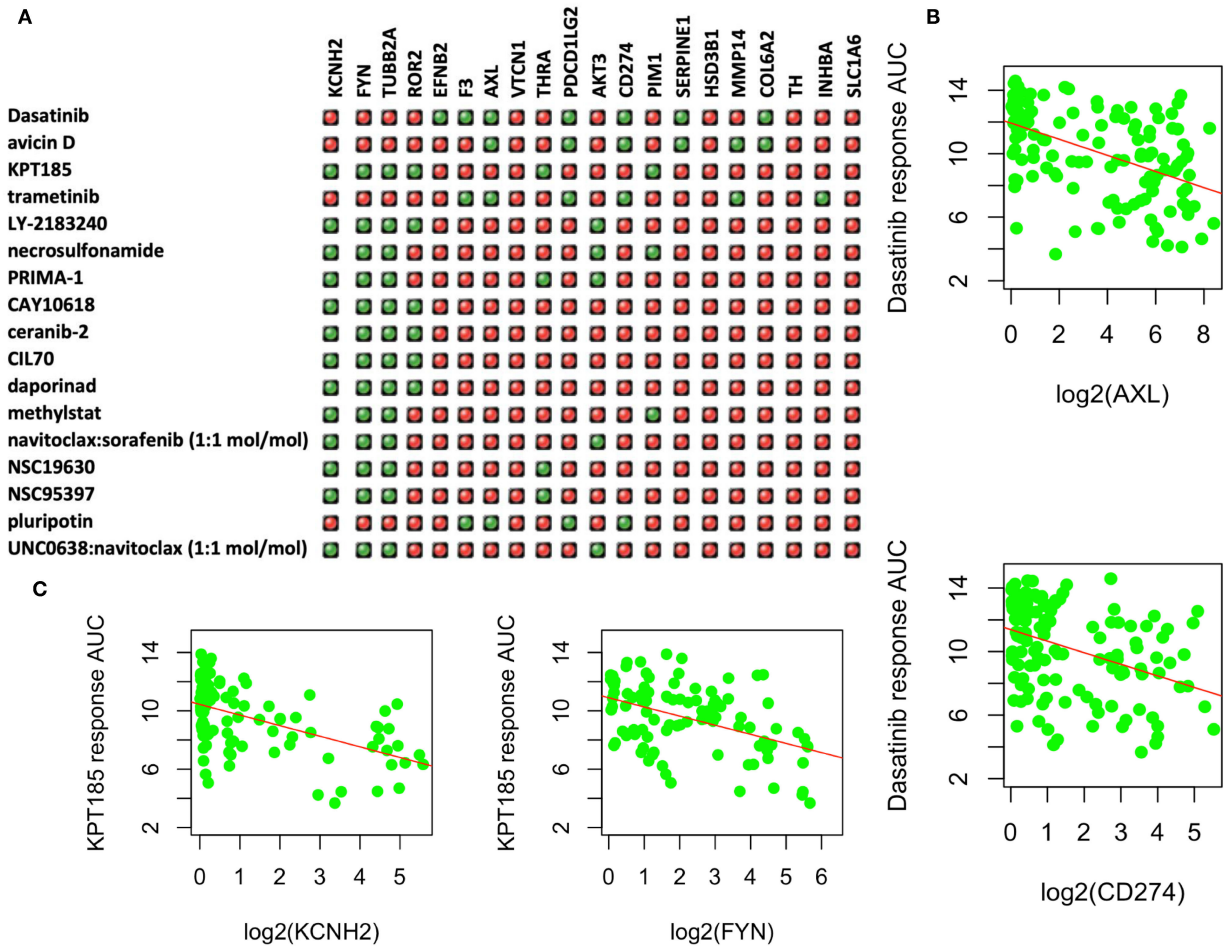
Upregulated genes and targeting drugs from combination analysis of our, CCLE, and CTRP data. **(A)** Top 17 drugs whose response is negatively correlated with at least four upregulated genes in PC9GR cells as indicated in green “light.” Red ones are those not significantly correlated pairs. **(B)** Scatterplot between Dasatinib response (*y*-axis, AUC% representing percentage of area under the curve) and gene expression of selected genes (*AXL* and *CD274* on *x*-axis, which is log2 RPKM value) in 142 lung cancer cell lines of CCLE. Higher expression of both genes is significantly correlated with better response (smaller area under the curve or AUC). **(C)** Scatterplot between KPT-185 response (*y*-axis, AUC% representing percentage of area under the curve) and gene expression of selected genes (*KCHN2* and *FYN* on *x*-axis, which is log2 RPKM value) in 142 lung cancer cell lines of CCLE. Higher expression of both genes is significantly correlated with better response (smaller area under the curve or AUC).

Among the upregulated DEGs, 31 had known available drugs potentially targeting these genes (Table S2). We analyzed the expression correlation of these genes with drug response profiles using CCLE and CTRP data sets for 144 lung cancer cell lines. Among 31 genes, we found 20 genes with at least one drug significantly correlated with drug responsiveness (*p* < 0.001 and correlation *R* <-0.3) for 160 out of 545 drugs. We then focused on these drugs whose response was negatively correlated with at least four of these genes as shown in Figure 4A. Most of these drugs are used for other cancers or diseases. For example, Dasatinib is a selective tyrosine kinase receptor inhibitor being used to treat chronic myelogenous leukemia (CML) positive for the Philadelphia chromosome. The expression of seven genes (out of 20 upregulated DEGs in PC9GR cells) was correlated with the response to this drug, and administration of the drug may reverse cell resistance to gefibinib as an alternative treatment. Among the negatively correlated genes, *AXL* expression is known to be associated with TKI resistance and is emerging as a new treatment target (40, 41). Higher expression of this gene was correlated with a better response to Dasatinib in lung cancer cell lines (Figure 4B). CD274 (*PD-L1*) is an adaptive immune response suppressor and blockade of PD1/PD-L1 represents a major cancer treatment breakthrough. Similarly, higher expression of *CD274* was an indicator for better response to Dasatinib (Figure 4B). As another example, KPT-185 is a *CRM1* inhibitor, a protein that mediates the nuclear export of proteins, rRNA, snRNA, and some mRNAs. The expression of six upregulated genes in PC9GR cells (Figure 4A) was significantly correlated with the response to KPT-185, such as *KCNH2* and *FYN* (Figure 4C).

### Gefitinib-Resistant PC9GR Cells Respond to Dasatinib, KPT-185, and Trametinib

**Figure 5:**
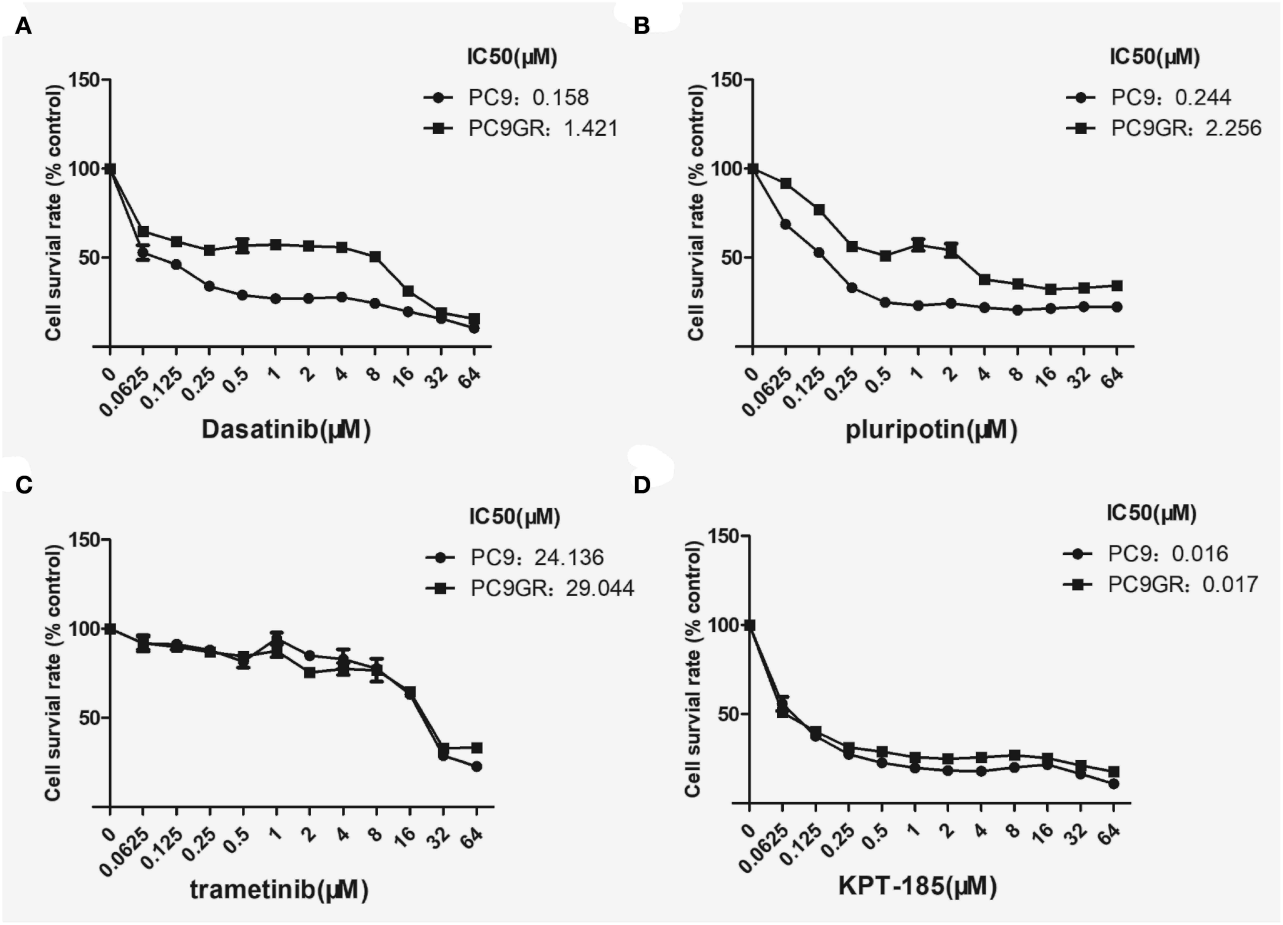
Dose and response curves of PC9 and PC9GR cells to Dasatinib, plurpotin, trametinib, and KPT-185. PC9 and PC9GR cell are treated with Dasatinib **(A)**, plurpotin **(B)**, trametinib **(C)**, and KPT-185 **(D)** at doubling dose for 72 h. Cell viability (*y*-axis) is detected by CCK-8 assay. KPT-185 is the strongest inhibitor for both PC9 and PC9GR with IC50 0.016 and 0.017, respectively.

**Figure 6:**
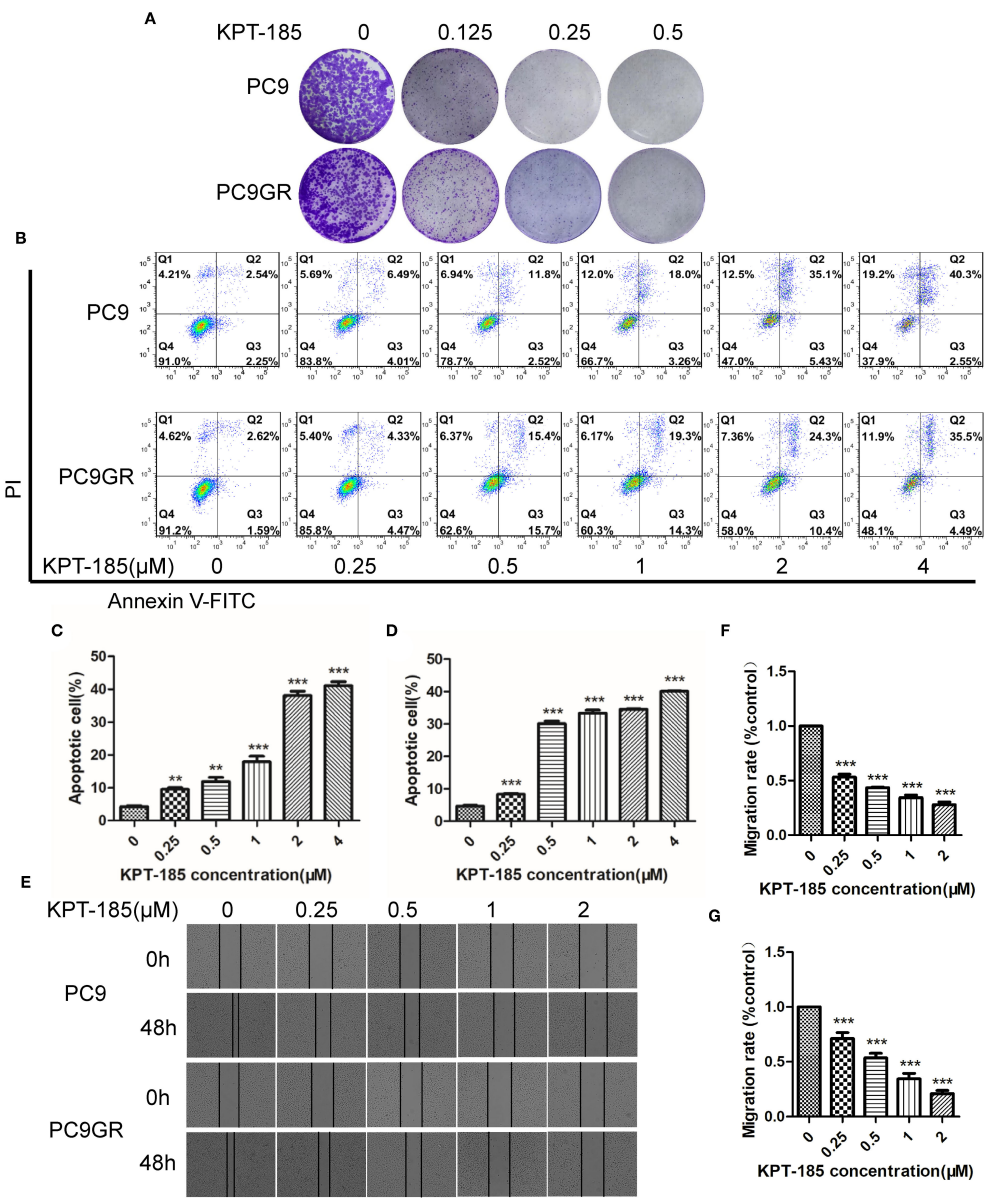
The proliferation inhibition, apoptotic induction, and antimigration effect of KPT-185 on PC9 and PC9GR cells. **(A)** The representative images of colony formation by PC9 and PC9GR cells after being treated with KPT-185 at 0, 0.125, 0.25, 0.5 μM for 9 days. The inhibition is dose dependent and similar between PC9 and PC9GR cells. **(B)** Quadrant graphs of cell apoptosis analysis for PC9 and PC9GR after being treated with KPT-185 at five concentrations for 48 h using flow cytometry. X-axis represents Annexin V-FITC, and y-axis represents PI. Q1, Q2, Q3, and Q4, respectively, represent dead cell fragments, late apoptosis, early apoptosis, and normal cell community. **(C)** The ratios of apoptotic cells at different concentrations in PC9 cells. **(D)** The ratios of apoptotic cells at different concentrations in PC9GR cells. **(E)** The representative images of migration inhibition after KPT-185 treatments in PC9 and PC9GR cells by wound-healing assay (magnification at 100X). **(F)** The bar plot of migration inhibition rates at 0, 0.25, 0.5, 1, and 2 uM in PC9 cells. **(G)** The bar plot of migration inhibition rates at 0, 0.25, 0.5, 1, and 2 uM in PC9GR cells. The bar height represents mean ± SEM of three independent experiments (*n* = 3, **P < 0.01, ***P < 0.001 compared to controls).

Among the top five drugs whose response was correlated with the highest number of upregulated genes in the CCLE lung cancer cell lines, Dasatinib, KPT-185, and trametinib are available to purchase for evaluation (Avicin D is an extracted chemical from plants, and LY2183240 is a potent inhibitor of Cannabinoid absorption). We also added pluripotin as its response was associated with expression of *AXL* and *CD274*. As shown in Figure 5, all four drugs except trametinib demonstrated significant inhibition effects on both PC9 and PC9GR cells although PC9 cells generally were more sensitive. However, the effect of KPT-185 on resistant PC9GR cells was almost the same or better than on PC9 cells. Further experiments on KPT-185 showed that it significantly suppressed cell growth (Figure 6A) and induced apoptosis of PC9GR cells at the similar or better rates (at lower concentrations, Figure S1) as the PC9 cells in a concentration-dependent manner (Figures 6B–D). KPT-185 also significantly reduced the cell migration of PC9 and PC9GR cells (Figures 6E–G).

## Discussion

Lung adenocarcinoma with *EGFR* mutations (primarily exon 19 deletions and L858R substitution in exon 21) is sensitive to targeted therapy by TKIs, such as gefitinib and erlotinib, which offers significant survival benefit to patients. Such therapy is better tolerated than traditional chemotherapies. However, eventually developed resistance to these drugs is a major clinical challenge. Although the majority of these resistant tumors are explained by subsequent T790M mutation, how this mutation causes resistance and what other genomic abnormalities occur are not well-characterized. Understanding of the mechanisms helps identify new targets and develop new therapeutic strategies.

In this study, we profiled the transcriptomes before and after PC9 lung cancer cells obtained resistance to gefitinib with development of traditional T790M mutation and found that over 20% of genes (6,699 out of 31,290) were highly differentially expressed at stringent criteria. T790M is considered as a gatekeeper mutation, which does not directly block inhibitor binding to the active site of *EGFR* but increases its affinity for ATP so that a TKI is outcompeted for its reduced anti-EGFR activities. The dramatically changed transcriptome suggests the resistant mechanism of PC9GR cells was far more complex, and the combination of additional mutations and long exposure to TKIs may led to PC9GR cells' transcriptome reprograming and metabolic changes to adapt their survival. Multiple pathways were changed, and the most noticeable ones were TNFA_signaling_via_NFKB, androgen/estrogen response, P53_pathway, MTORC1_signaling, and hypoxia and epithelial_mesenchymal_transition. NF-κB activation was observed as an early event of resistance development with increased expression of *NFKB1* (42). Both TNF and hypoxia were related to adaptive resistance of non-small cell lung cancer cells as reported previously (43). Epithelial-to-mesenchymal transition is also known to be involved in late obtained T790M-associated drug-resistant PC9 cells (PC9GR3) (38).

Our search for new treatment targets found that several existing drugs used for other cancers or diseases may be repurposed to treat those lung cancer patients with acquired resistance to gefitinib. Dasatinib is a selective tyrosine kinase receptor inhibitor used to treat CML. Recent studies have shown that lung cancer with increased *YES1* was more responsive to Dasatinib (44), and increased expression of *YES1* was reported to be one of the acquired resistant mechanisms to *EGFR* inhibitors in lung cancer with *EGFR* mutations (45). Our data is consistent with the reports as *YES1* was upregulated in PC9GR cells by 1.5-fold, and these resistant cells were responsive to Dasatinib. KPT-185 is a *CRM1* inhibitor, a protein that mediates the nuclear export of proteins, rRNA, snRNA, and some mRNAs. KPT-185 significantly inhibits cancer cell proliferation and induces cell-cycle arrest and apoptosis, such as cancer cells from acute myeloid leukemia (46), pancreatic cancer (47) and NSCLC, including EGFR-TKI-resistant cell lines (48). Our data showed that KPT-185 could inhibit proliferation and induce apoptosis of both PC9GR and PC9 cells yet had a stronger effect causing apoptosis of PC9GR cells at lower concentrations of 0.5 and 1 μM (Figure S1), which can be important as a loose dose is less toxic and desired for human administration.

In addition to KPT-185, there are several other CRM1 inhibitors in various phases of development and applications, which include KPT-249, KPT-251, KPT-276, KPT-330 (Selinexor), KPT-335 (Verdinexor), and KPT-8602 (Eltanexor) (https://www.selleckchem.com/crm1.html) (49). These small-molecule drugs are selective inhibitors of nuclear export (SINEs) that covalently bind to Cysteine 528 residue of CRM1 in a slowly reversible fashion for their anticancer activities. Preclinical and clinical studies have demonstrated their efficacies on a number of solid and hematologic cancers (50). Although our study focused on KPT-185 as it is the drug with response profiles in the CRTP database, the results or conclusions may be applicable to other drugs in the family considering their similar mechanism of actions. In fact, for clinical application, a drug such as KPT-330 may be preferable because of its better bioavailability, mild side effects, and approval for clinical usage (relapsed refractory multiple myeloma). Studies also demonstrate its synergistic effect with cisplatin (51). A multicenter phase I/II clinical trial of selinexor with docetaxel in treating KRAS-mutant NSCLC is being conducted (https://clinicaltrials.gov/ct2/show/NCT03095612). A combination of CRM1 inhibitors with EGFR-TKIs or other drugs as a third- and fourth-line treatment warrants further investigation for TKI-resistant tumors.

Pluripotin is a small molecule that inhibits ERK1 MAPK3 and RasGAP. It also inhibits RSK1, RSK2, RSK3, and RSK4 with IC50s of 0.5, 2.5, 3.3, and 10.0 μM, respectively (52). *MAPK3* was increased more than 1.6-fold in PC9GR cells while *RASA1* had no differential expression. In CCLE lung cell line data, pluripotin's response was associated with the expression of *F3, AXL, PDCD1LG2*, and *CD274*, and these genes were highly expressed in PC9GR cells. Although its inhibitory effect for PC9GR cells was not as strong as for PC9 cells, it had a synergistic effect when combined with gefitinib (Figure S2). Further evaluation of its combination may clarify its potential.

For gene expression, our data recapitulated some previous reports. *AXL* was significantly increased, and *MAPK1, VIM, GAS6, NFKB1, and KDM5A* were slightly increased in our data. Interleukin-6 was identified as an important factor in hypoxia- and aldehyde dehydrogenase-based gefitinib adaptive resistance in non-small cell lung cancer cells (43). Our data also show that the hypoxia pathway was significantly enriched, and *IL6* had about a 2-fold increase in PC9GR cells. On the other hand, some genes reported no change in PC9GR cells. For example, *NFKB1* is reported to be upregulated as an early event of TKI resistance (42); however, for the cells with long exposure to gefitinib with stable resistance, this gene was not activated and was significantly decreased more than 1.5-fold in PC9GR cells. *FGF2* was not significantly changed although *FGFR1* was increased about 1.8-fold (39). The discrepancies could be the results of tumor heterogeneities and experimental conditions. Literature reports are from different groups and different sets of tumors or cell lines. For the same PC9 cell line, our resistance line was established after long-term (12 months) exposure to gefitinib while other groups profiled resistant cells established at 1.5 or 6 months (38).

Our study found several interesting genes not reported in TKI-resistant cells before. *BASP1* was one of the most upregulated genes in PC9GR cells, yet its functions are largely unknown. Some studies have showed it is a tumor suppressor, and restoration of its expression in cancer cells inhibits tumor growth and migration for thyroid cancer (53). High expression of this gene is associated with better patient survival in breast cancer (54) and pancreatic cancer in which better response to adjuvant chemotherapy is also observed (55). On the other hand, studies found that this gene promoted cancer growth and was a poor prognostic factor in cervical cancer (56). The roles and functions of *BASP1* are likely cancer type and context specific. *COL6A2* is another gene significantly upregulated in PC9GR cells, and previous reports showed that increased expression of multiple collagen genes, including *COL6A2* was associated with drug resistance, tumor metastasis, and poor patient survival in ovarian cancer (57, 58).

Mechanisms of resistance to TKIs are complicated and multifold (59). Through gene expression profiling, a previous study reported the increased activity of autophagy mediated by *ERK* and *AKT* activation in resistant cells (60). Cellular proliferation, apoptosis, and cell cycle were also involved (61). Although our study has overlap with these reports, differences exist, such as some top regulated genes reported had no change or opposite changes in our study (*CDKN2B* was found to be up-expressed, but it was down in resistant cells in our study). These differences highlight the heterogeneity of cell lines, and resistant mechanisms may diverge according to exposure conditions.

Because of these complexities, clinical management for resistance tumors needs a combination strategy targeting multiple pathways or genes. Chemo-drug combinations can have a synergistic effect. For example, we tried the combination of gefitinib with either dasatinib or pluripotin, and both showed synergistic effects at certain concentration combinations with combination index (CI) <1 (Figure S2). We did not evaluate the synergistic effect of KPT-185 as it already had a very strong inhibitory effect on PC9GR cells; however, its combination with other drugs might even be more effective in the treatment of EGFR-TKI-resistant lung cancer, which is one of our future plans.

In spite of the interesting findings, our conclusions are from a single lung cancer cell line, which may or may not be generalizable to other cell lines. Future studies need to include more cell lines with different mutation status. Additionally, our drug response data are limited to *in vitro* cell lines, and studies are needed *in vivo* or clinical trials to translate the findings.

## Conclusions

In conclusion, our study shows that acquired TKI-resistant lung cancer cells (PC9GR) have significantly altered the transcription and pathway regulation for new therapeutic targets. Existing drugs may be reused to treat patients who are resistant to TKIs.

## Data Availability Statement

The original contributions presented in the study are publicly available. This data can be found here: the NCBI Gene Expression Omnibus (GSE129221).

## Author Contributions

NW and ZS contributed to the study design, data analysis, and manuscript writing. YS and FZ contributed to materials and analysis. XZ contributed to the design and revised the manuscript critically. All authors read and approved the final manuscript.

## Conflict of Interest

The authors declare that the research was conducted in the absence of any commercial or financial relationships that could be construed as a potential conflict of interest.

## Supplementary Material

Figure S1 Apoptotic effect of KPT-185 on PC9 and PC9GR.

Figure S2 Inhibition effects of drug combinations.

Table S1 Differentially expressed genes (*n* = 864) between resistant and sensitive lung cancer cells.

Table S2 Upregulated genes in resistant cells and their available targeting drugs.

## Abbreviations

*EGFR*: epidermal growth factor receptor
TKIs: tyrosine kinase inhibitors
CCLE: cancer cell line encyclopedia
CTRP: Cancer Therapeutics Response Portal
NSCLC: non-small cell lung cancer
EMT: epithelial-to-mesenchymal transition
RNA-seq: RNA sequencing
FDR: false discovery rate
FPKM: fragments per kilobase million
DEGs: differentially expressed genes
RITAN: rapid integration of term annotation
STAR: spliced transcripts alignment to a reference
IPA: ingenuity pathway analysis
RPKM: reads per kilobase million
GEO: gene expression omnibus
IC50: half maximal inhibitory concentration
PCA: principal components analysis
KEGG: kyoto encyclopedia of genes and genomes
CML: chronic myelogenous leukemia.
