# Supplementary material for: Transcriptome Profiling of Acquired Gefitinib Resistant Lung Cancer Cells Reveals Dramatically Changed Transcription Programs and New Treatment Targets
Source: Front Oncol. 2020 Aug 14;10:1424. doi: 10.3389/fonc.2020.01424 (PMC7456826; doi:10.3389/fonc.2020.01424)
Supplement: Figure S1 — Apoptotic effect of KPT-185 on PC9 and PC9GR. [file Image_1.PDF]

Figure S1. Apoptotic effect of KPT-185 on PC9 and PC9GR

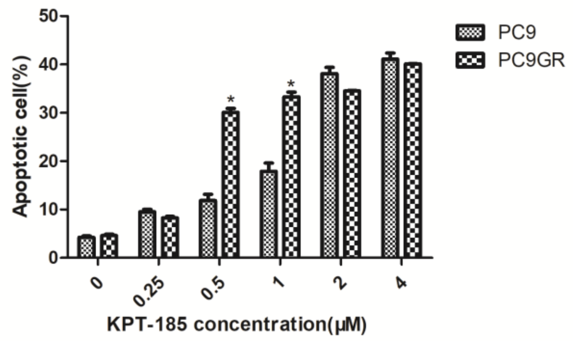

Comparison of KPT-185 induced apoptotic effects on PC9 and PC9GR. KPT-185 had stronger apoptotic effect on PC9GR cells at lower concentrations compared to PC9 cells (P value 0.0127 and 0.0287 at 0.5 and 1 μ M respectively).

Figure S2: Inhibition effects of drug combinations

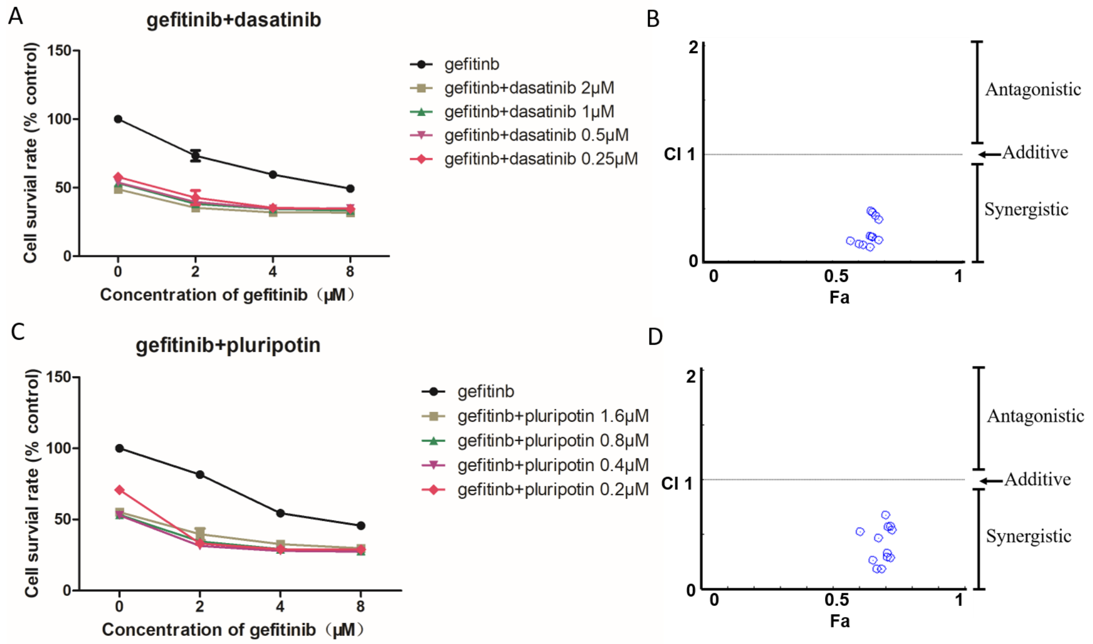

Synergistic effect of gefitinib with either dasatinib or pluripotin. (A) The combination of different concentrations of gefitinib(0μM、2μM、4μM、8μM) and dasatinib(0μM、0.25μM、 0.5μM、 1μM、 2μM) on PC9GR cells significantly reduced cell viability; (B) The CIs of gefitinib and dasatinib are less than 1 by CompuSyn software; (C) The combination of different concentrations of gefitinib(0μM、2μM、4μM、8μM) and pluripotin(0μM、0.2μM、0.4μM、0.μM、1.6μM) on PC9GR cells significantly reduced cell viability; (D) The CIs of gefitinib and pluripotin are also ess than 1.
